# Supplementary material for: YAP and TAZ couple osteoblast precursor mobilization to angiogenesis and mechanoregulated bone development
Source: bioRxiv. 2023 Jan 21:2023.01.20.524918. Preprint. [Version 1] doi: 10.1101/2023.01.20.524918 (PMC9882292; doi:10.1101/2023.01.20.524918)
Supplement: Supplement 1 [file NIHPP2023.01.20.524918v1-supplement-1.pdf]

bioRxiv preprint doi: https://doi.org/10.1101/2023.01.20.524918; this version posted January 21, 2023. The copyright holder for this preprint (which was not certified by peer review) is the author/funder, who has granted bioRxiv a license to display the preprint in perpetuity. It is made available under aCC-BY 4.0 International license.

Supplementary figures

Figure S1. Additional data on the Osx-conditional YAP/TAZ knockout humeri at E17.5.
Figure S2. E15.5 and E17.5 Col2-CFP; ColX-RFP; Col1(3.6)-YFP intensity line plots by individual channel.
Figure S3. Osx::GFP^{+} cell density in E17.5 humeri.
Figure S4. Phalloidin stained E14.5 and E15.5 WT$^{fl/fl}$ and YAP/TAZ cKO^{Osx}.
Figure S5. Cell clusters in the whole limb merged into major cell types.
Figure S6. Major cell types in the fetal forelimb.
Figure S7. Osteoblast cell states.
Figure S8. Gene plot visualization of gene expression in WT^{Osx}, WT$^{fl/fl}$, YAP/TAZ cKO^{Osx} in the osteoblast cell states.
Figure S9. Supplemental data on the vessel associated osteoblast precursor (VOP) cell state.
Figure S10. Chondrocyte cell states.
Figure S11. Endothelial cell states.
Figure S12. Cell-cell communication between osteoblast cell type and endothelial cell type by CellChat.
Figure S13. Cell-cell communication between Vessel associated osteoblast precursors (VOPs) and type H endothelial cells by CellChat.
Figure S14. Osx::GFP+ cell proximity to Endomucin^{+} blood vessels and computational varied tests.
Figure S15. Quantification of the blood vessels within 50μm of the chondro-osseous junction in the core and outer annulus.
Figure S16. Bone collar diameter of explant C57Bl6 limbs in the cultured in the mechanostimulation bioreactor.
Figure S17. Supplemental data for the genetic Osx-conditional YAP/TAZ deletion bioreactor experiments.

bioRxiv preprint doi: https://doi.org/10.1101/2023.01.20.524918; this version posted January 21, 2023. The copyright holder for this preprint (which was not certified by peer review) is the author/funder, who has granted bioRxiv a license to display the preprint in perpetuity. It is made available under aCC-BY 4.0 International license.

Supplementary figures

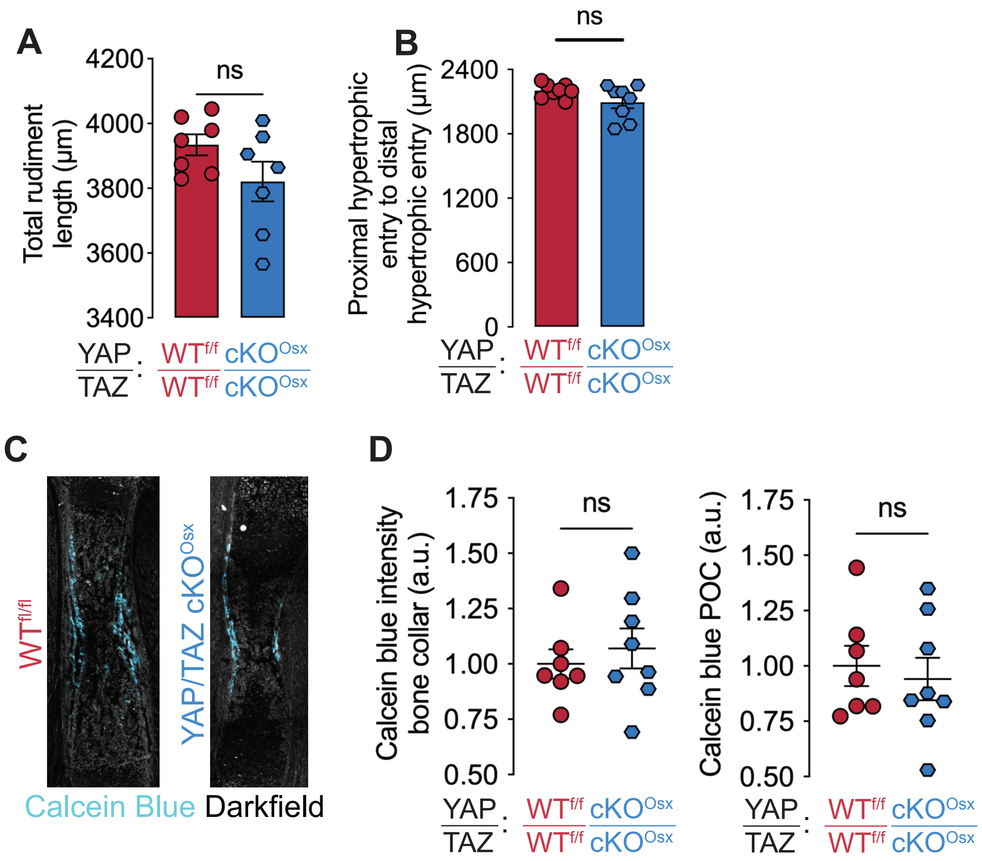

Figure S1. Additional data on the Osx-conditional YAP/TAZ knockout humeri at E17.5. (**A**) Length of the humerus rudiment at E17.5. (**B**) Distance between proliferating chondrocyte zones. i.e. the sum of both proximal and distal hypertrophic zones and primary ossification center at E17.5. (**C**) Calcein blue staining of E17.5 humerus. (**D**) Quantification of Calcein blue images in the bone collar and primary ossification center (POC).

bioRxiv preprint doi: https://doi.org/10.1101/2023.01.20.524918; this version posted January 21, 2023. The copyright holder for this preprint (which was not certified by peer review) is the author/funder, who has granted bioRxiv a license to display the preprint in perpetuity. It is made available under aCC-BY 4.0 International license.

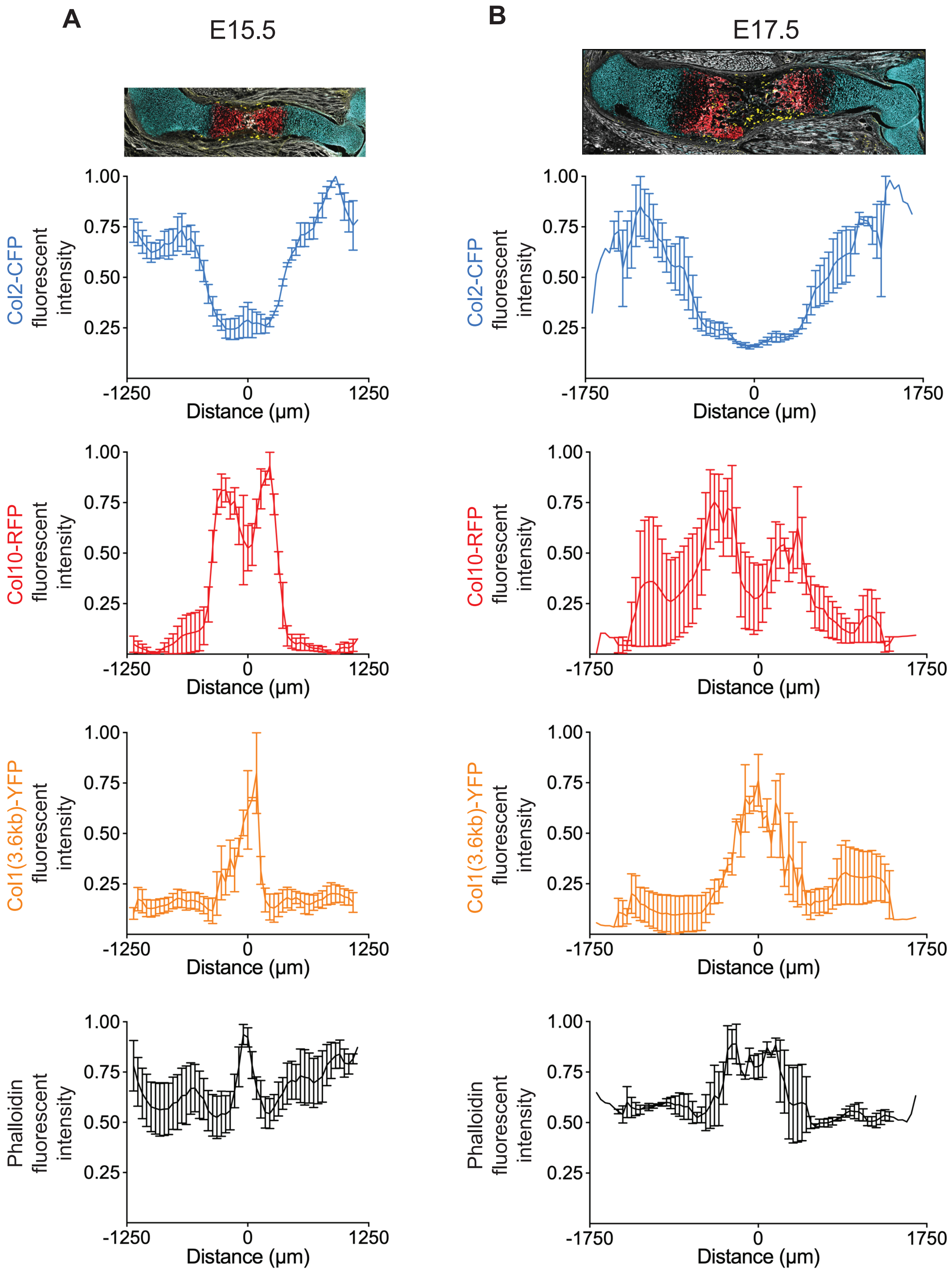

Figure S2. E15.5 and E17.5 Col2-CFP; ColX-RFP; Col1(3.6)-YFP intensity line plots by individual channel. (**A**) E15.5. (**B**) E17.5. Images are scaled to the plots. Images shown are repeated from figure 2 for clarity of the individual channel plots.

bioRxiv preprint doi: https://doi.org/10.1101/2023.01.20.524918; this version posted January 21, 2023. The copyright holder for this preprint (which was not certified by peer review) is the author/funder, who has granted bioRxiv a license to display the preprint in perpetuity. It is made available under aCC-BY 4.0 International license.

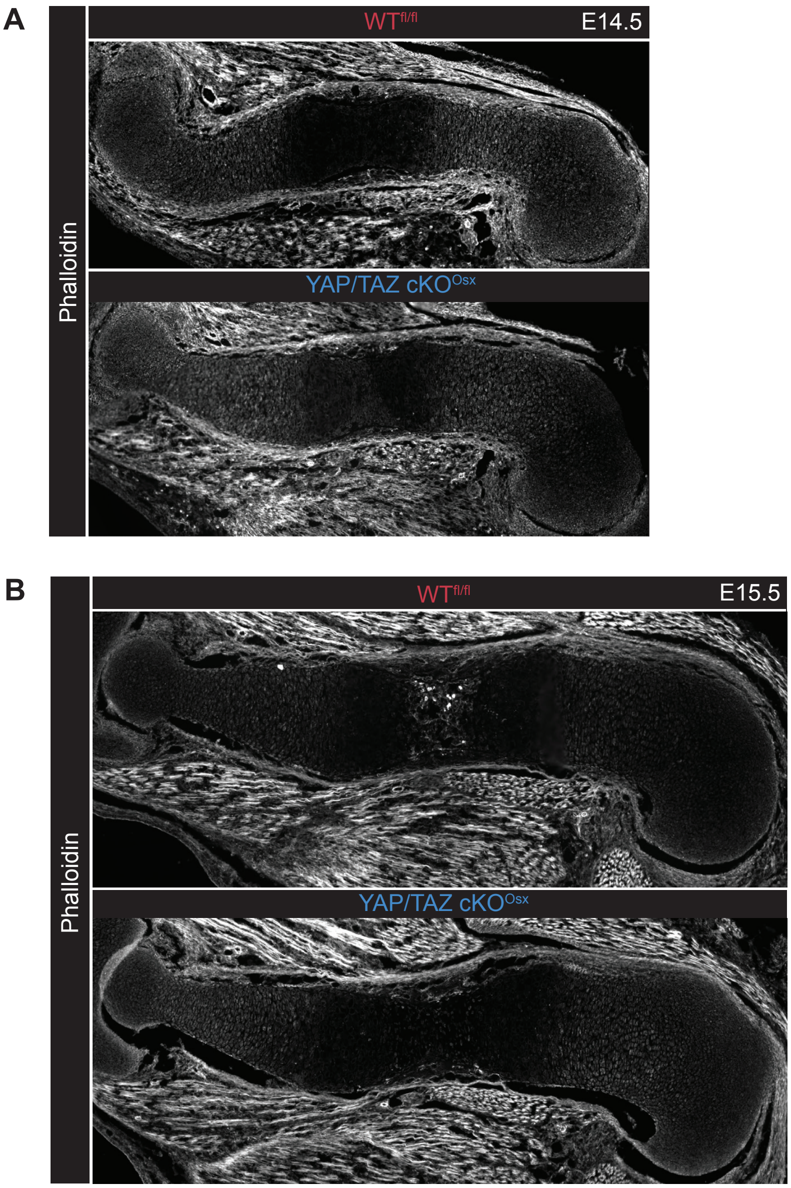

Figure S3. Phalloidin stained E14.5 and E15.5 WT$^{fl/fl}$ and YAP/TAZ cKO^{Osx}. (**A**) E14.5 humeri. (**B**) E15.5 humeri

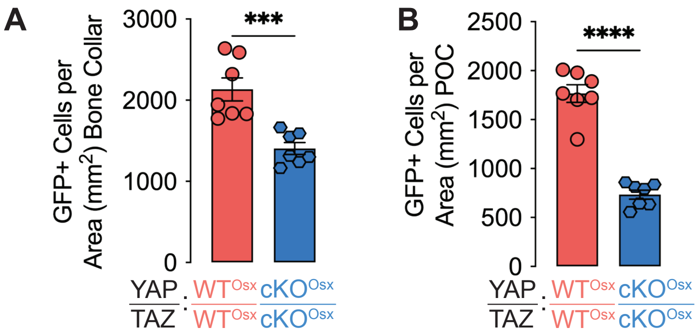

Figure S4. Osx::GFP^{+} cell density in E17.5 humeri. (**A**) Osx::GFP^{+} cell density in the bone collar. (**B**) Osx::GFP^{+} cell density in the primary ossficiation center (POC).

bioRxiv preprint doi: https://doi.org/10.1101/2023.01.20.524918; this version posted January 21, 2023. The copyright holder for this preprint (which was not certified by peer review) is the author/funder, who has granted bioRxiv a license to display the preprint in perpetuity. It is made available under aCC-BY 4.0 International license.

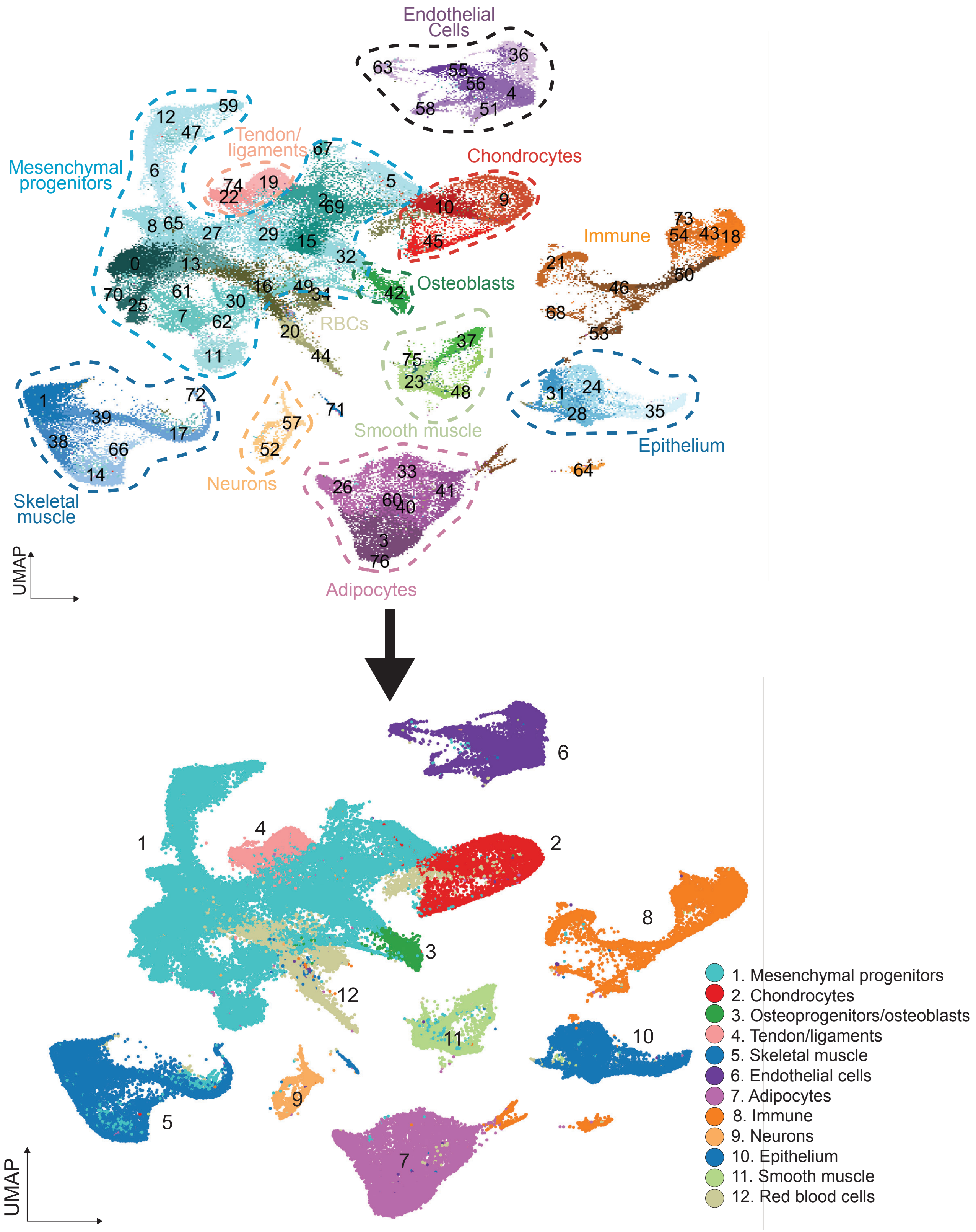

Figure S5. Cell clusters in the whole limb merged into major cell types. Louvain clustering identified 77 clusters in the whole fetal limb. We merged these clusters into 12 major cell types.

bioRxiv preprint doi: https://doi.org/10.1101/2023.01.20.524918; this version posted January 21, 2023. The copyright holder for this preprint (which was not certified by peer review) is the author/funder, who has granted bioRxiv a license to display the preprint in perpetuity. It is made available under aCC-BY 4.0 International license.

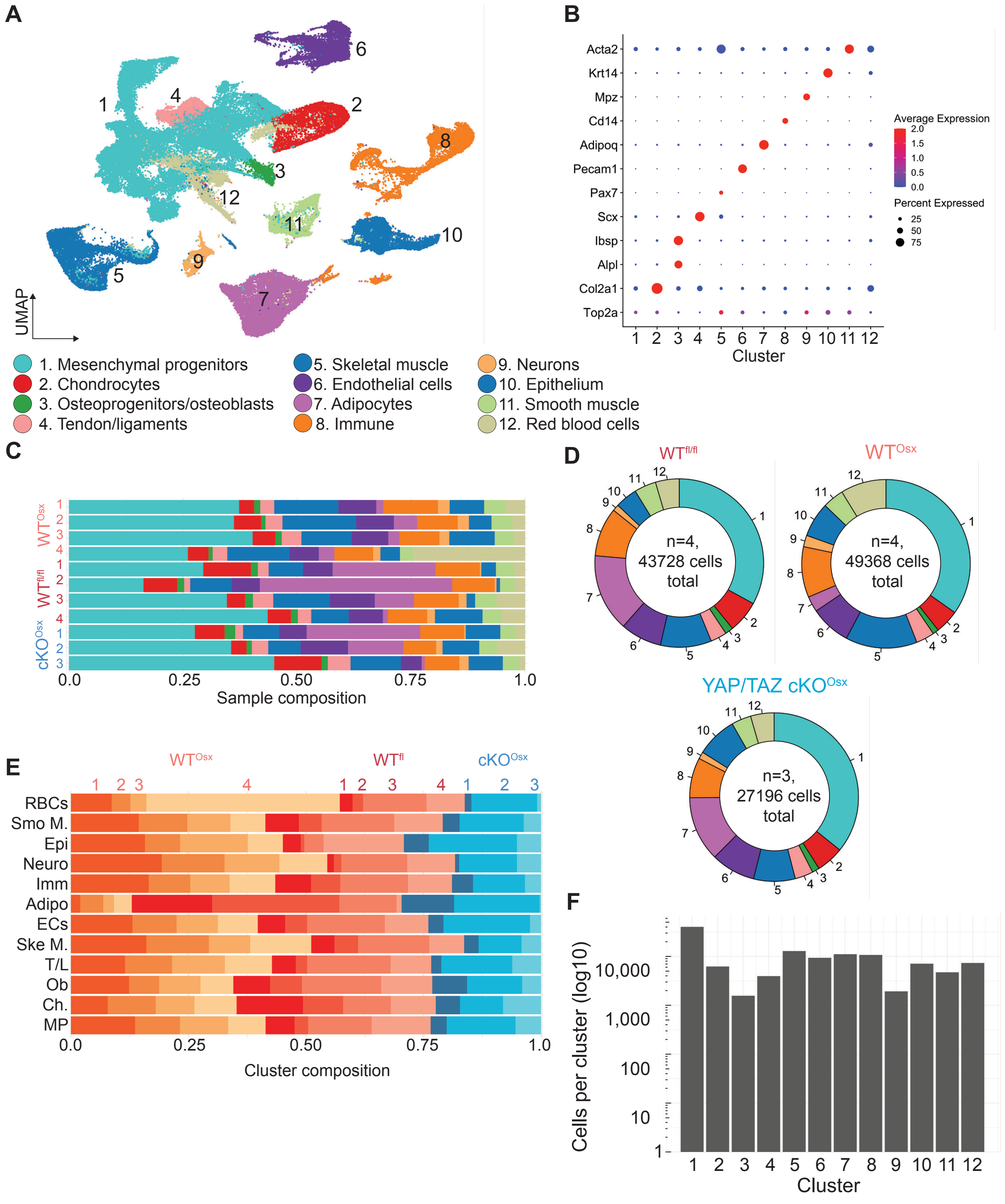

Figure S6. Major cell types in the fetal forelimb. (**A**) UMAP visualization showing cell types in from WT$^{fl/fl}$, WT^{Osx}, YAP/TAZ cKO^{Osx} fetal forelimbs. (**B**) Dotplot showing expression of canonical cell type markers. (**C**) Bar graph showing cell type proportions by sample. (**D**) Pie charts showing cell type proportions by genotype. The numbers in the middle of the pie chart indicates the genotype sample size and total high quality cells from each genotype analyzed here. (**E**) Bar graph showing sample proportions by cell type. (**F**) Bar graph showing the size of each major cell type cluster.

bioRxiv preprint doi: https://doi.org/10.1101/2023.01.20.524918; this version posted January 21, 2023. The copyright holder for this preprint (which was not certified by peer review) is the author/funder, who has granted bioRxiv a license to display the preprint in perpetuity. It is made available under aCC-BY 4.0 International license.

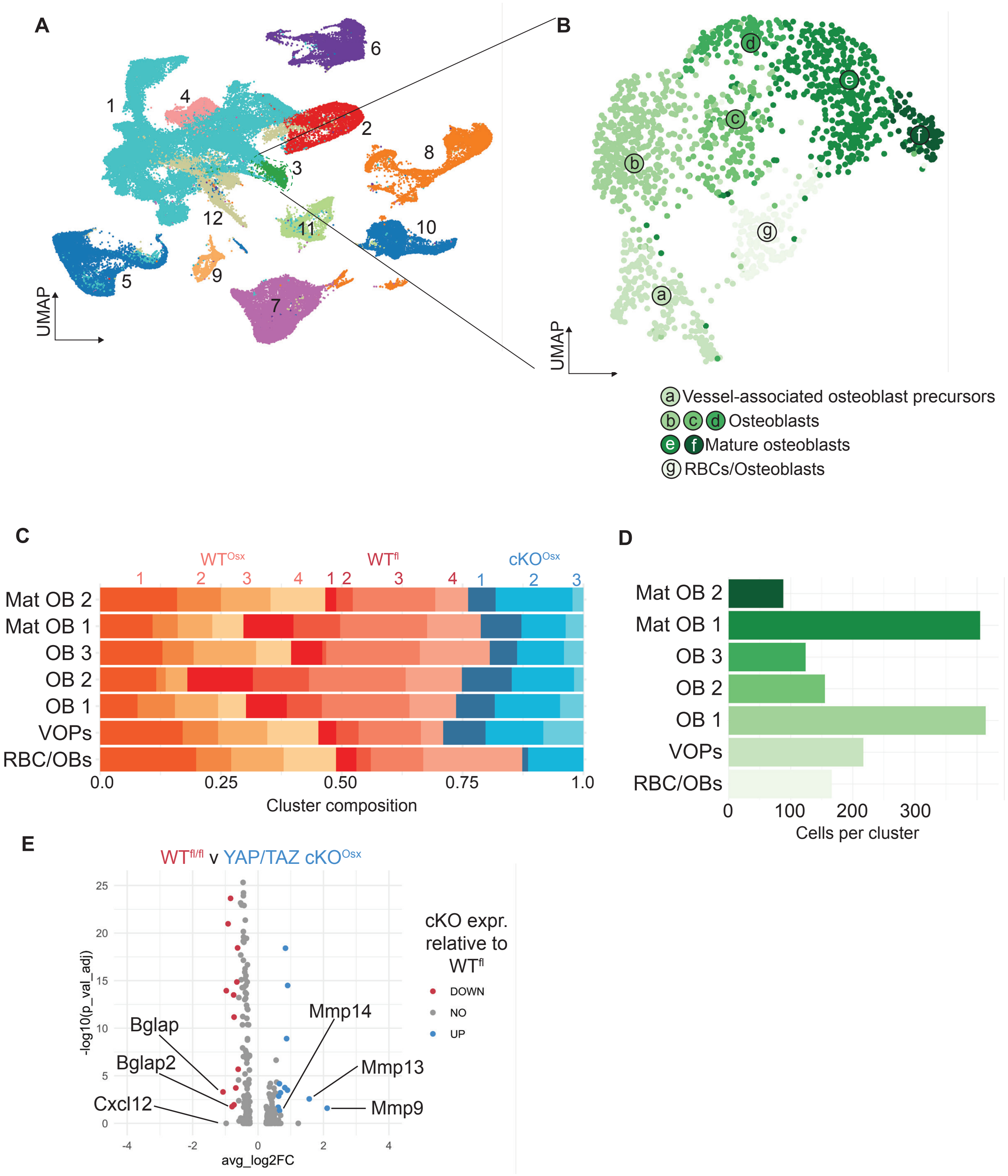

Figure S7. Osteoblast cell states. (**A**) UMAP visualization of fetal forelimb cells showing the osteoblasts highlighted. (**B**) UMAP visualization of osteoblast cell states. (**C**) Bar graph showing sample proportions by osteoblast cell state. (**D**) Bar graph showing the cluster size of each osteoblast cell state. (**E**) Volcano plot showing gene expression differences between $WT^{fl/fl}$ and YAP/TAZ cKO^{Osx} osteoblasts overall. Red is decreased in the YAP/TAZ cKO^{Osx}. Blue is increased in the YAP/TAZ cKO^{Osx}.

bioRxiv preprint doi: https://doi.org/10.1101/2023.01.20.524918; this version posted January 21, 2023. The copyright holder for this preprint (which was not certified by peer review) is the author/funder, who has granted bioRxiv a license to display the preprint in perpetuity. It is made available under aCC-BY 4.0 International license.

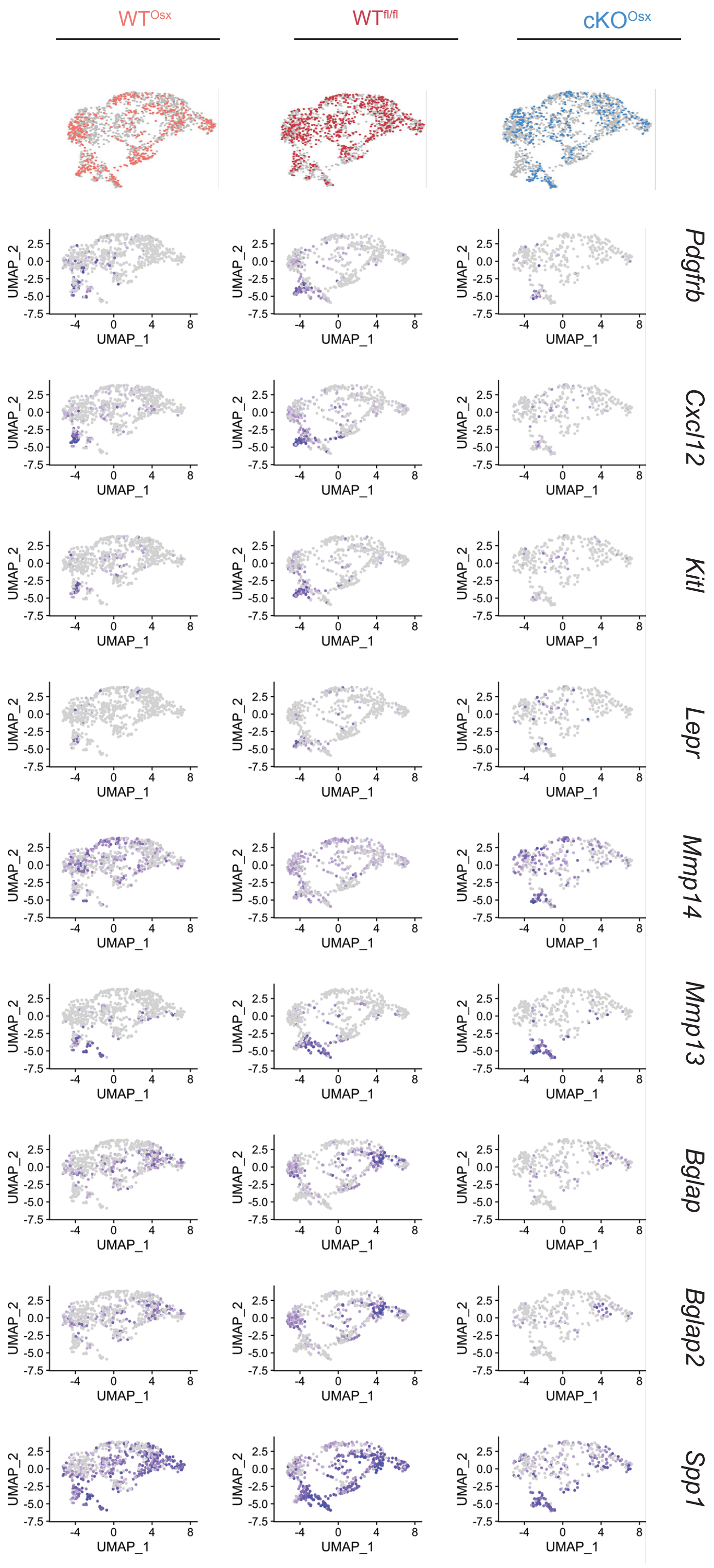

Figure S8. Gene plot visualization of gene expression in WT^{Osx}, WT$^{fl/fl}$, YAP/TAZ cKO^{Osx} in the osteoblast cell states. The top row shows the distribution of each genotype within the osteoblast cell states. The remaining rows show critically differentially expressed genes in the osteoblasts separated by genotype.

bioRxiv preprint doi: https://doi.org/10.1101/2023.01.20.524918; this version posted January 21, 2023. The copyright holder for this preprint (which was not certified by peer review) is the author/funder, who has granted bioRxiv a license to display the preprint in perpetuity. It is made available under aCC-BY 4.0 International license.

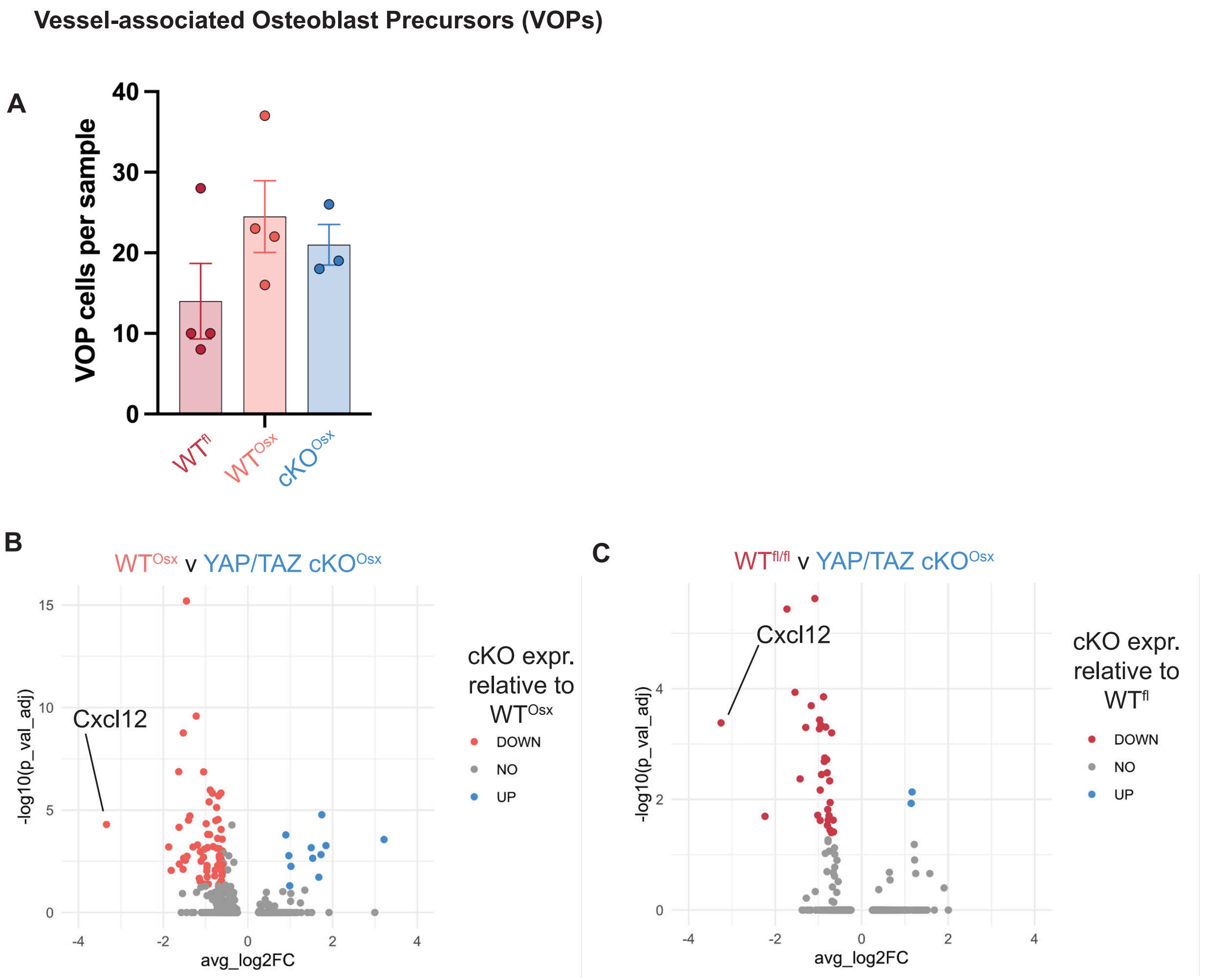

Figure S9. Supplemental data on the vessel associated osteoblast precursor (VOP) cell state. (**A**) Plot showing the number of VOPs in each sample, separated by genotype. (**B**) Volcano plot showing gene expression differences between WT^{Osx} and YAP/TAZ cKO^{Osx} VOPs. Red is decreased in the YAP/TAZ cKO^{Osx}. Blue is increased in the YAP/TAZ cKO^{Osx}. (**C**) Volcano plot showing gene expression differences between WT$^{fl/fl}$ and YAP/TAZ cKO^{Osx} VOPs. Red is decreased in the YAP/TAZ cKO^{Osx}. Blue is increased in the YAP/TAZ cKO^{Osx}. *Cxcl12* is the most differentially reduced gene when YAP/TAZ is deleted from VOPs in comparison to both Wildtypes.

bioRxiv preprint doi: https://doi.org/10.1101/2023.01.20.524918; this version posted January 21, 2023. The copyright holder for this preprint (which was not certified by peer review) is the author/funder, who has granted bioRxiv a license to display the preprint in perpetuity. It is made available under aCC-BY 4.0 International license.

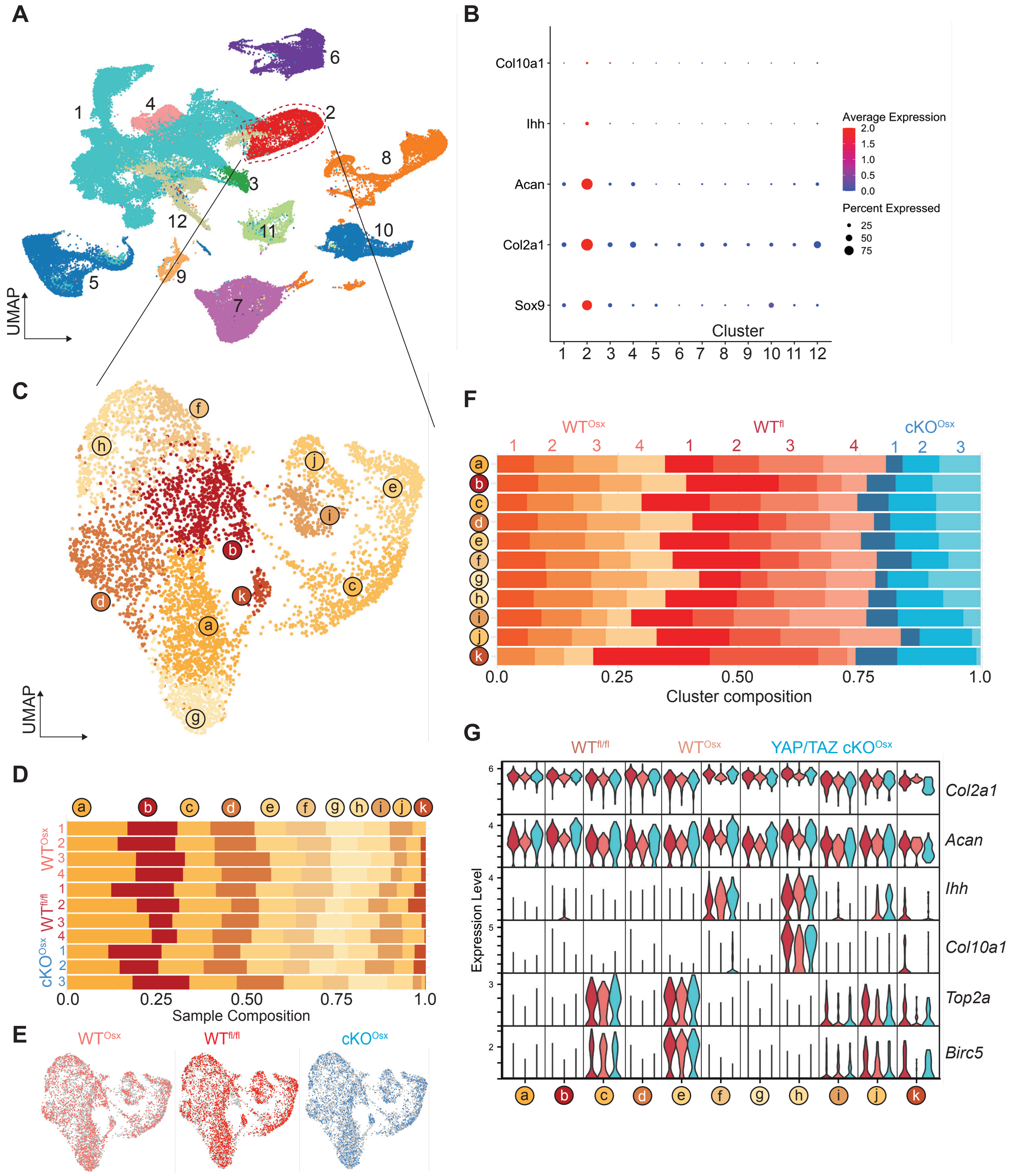

Figure S10. Chondrocyte cell states. (**A**) UMAP visualization of fetal forelimb cells showing the chondrocyte highlighted. (**B**) Dotplot showing chondrocyte marker gene expression among the whole fetal limb cell types (**C**) UMAP visualization of chondrocyte cell states. (**D**) Bar graph showing chondrocyte cell state proportions by sample. (**E**) UMAP visualization of chondrocyte cell states with each genotype highlighted. (**F**) Bar graph showing sample proportions by chondrocyte cell state. (**G**) Violin plot showing selected gene expression for each chondrocyte cell state by genotype.

bioRxiv preprint doi: https://doi.org/10.1101/2023.01.20.524918; this version posted January 21, 2023. The copyright holder for this preprint (which was not certified by peer review) is the author/funder, who has granted bioRxiv a license to display the preprint in perpetuity. It is made available under aCC-BY 4.0 International license.

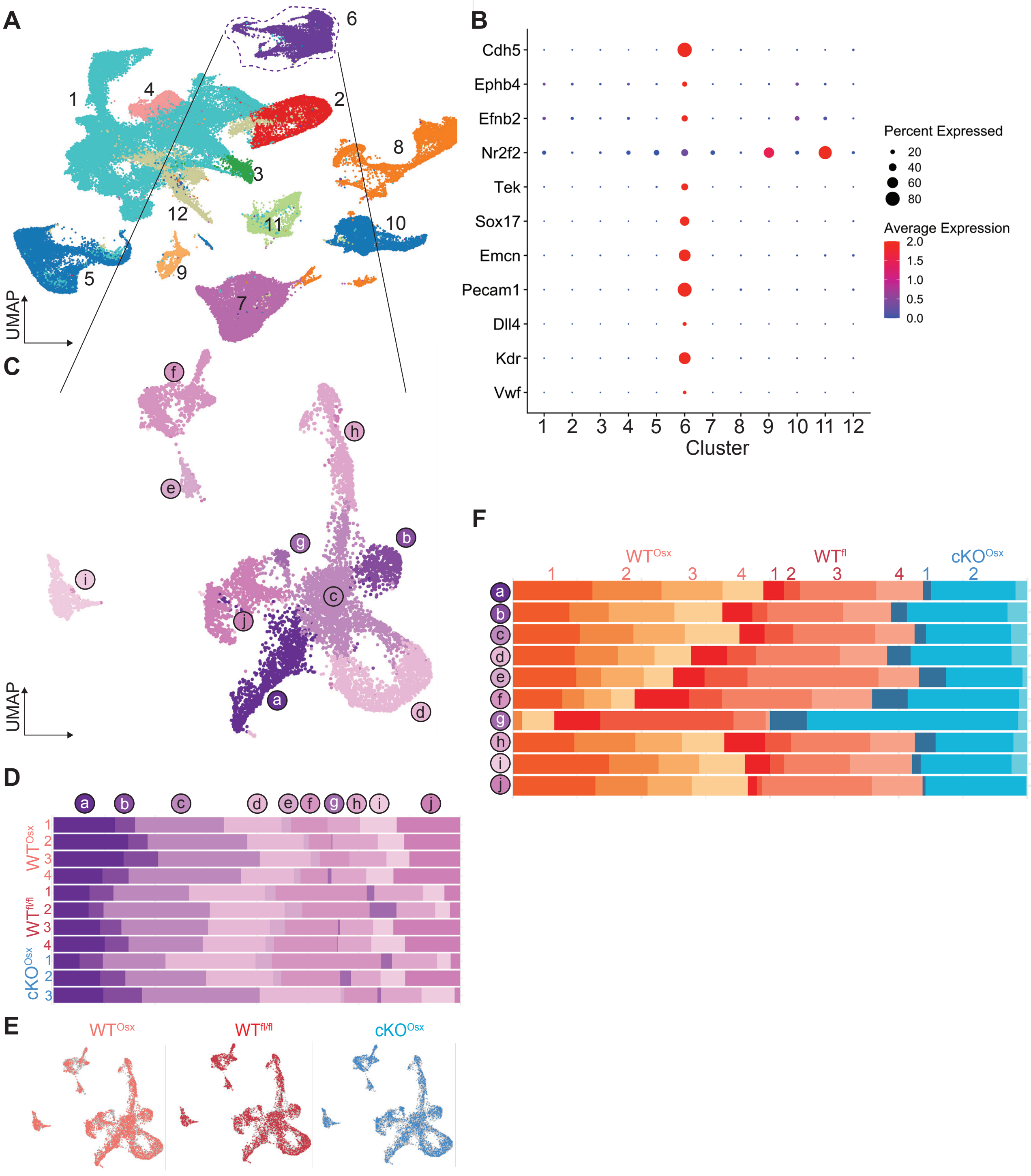

Figure S11. Endothelial cell states. (**A**) UMAP visualization of fetal forelimb cells showing the endothelial cells highlighted. (**B**) Dotplot showing endothelial marker gene expression among the whole fetal limb cell types (**C**) UMAP visualization of endothelial cell states. (**D**) Bar graph showing endothelial cell state proportions by sample. (**E**) UMAP visualization of endothelial cell states with each genotype highlighted. (**F**) Bar graph showing sample proportions by endothelial cell state.

bioRxiv preprint doi: https://doi.org/10.1101/2023.01.20.524918; this version posted January 21, 2023. The copyright holder for this preprint (which was not certified by peer review) is the author/funder, who has granted bioRxiv a license to display the preprint in perpetuity. It is made available under aCC-BY 4.0 International license.

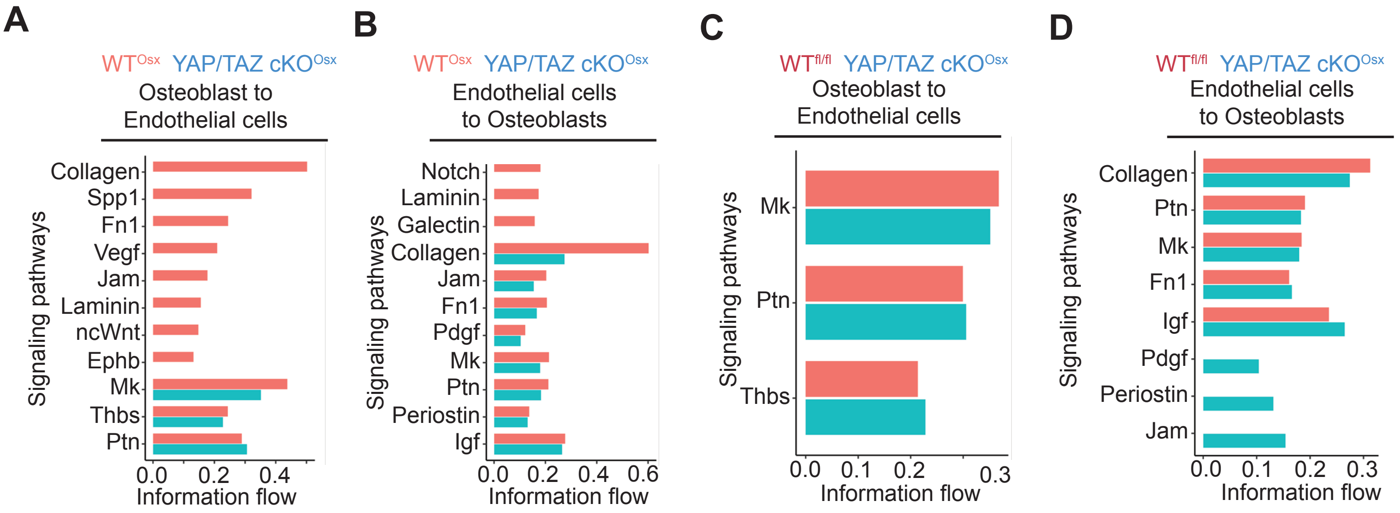

Figure S12. Cell-cell communication between osteoblast cell type and endothelial cell type by CellChat. (**A**) Cell-cell communication pathways from osteoblasts to endothelial cells in WT^{Osx} and YAP/TAZ cKO^{Osx}. (**B**) Cell-cell communication pathways from endothelial cells to osteoblasts in WT^{Osx} and YAP/TAZ cKO$^{Osx.}$ (**C**) Cell-cell communication pathways from osteoblasts to endothelial cells in WT$^{fl/fl}$ and YAP/TAZ cKO^{Osx}. (**D**) Cell-cell communication pathways from endothelial cells to osteoblasts in WT$^{fl/fl}$ and YAP/TAZ cKO^{Osx}.

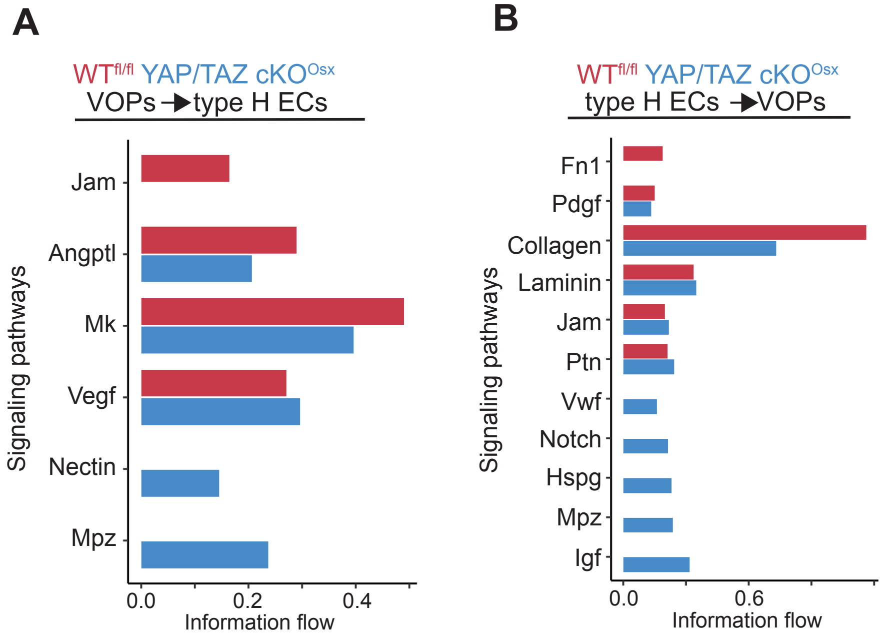

Figure S13. Cell-cell communication between Vessel associated osteoblast precursors (VOPs) and type H endothelial cells by CellChat. (**A**) Cell-cell communication pathways from VOPs to type H endothelial cells in WT$^{fl/fl}$ and YAP/TAZ cKO^{Osx}. (**B**) Cell-cell communication pathways from type H endothelial cells to VOPs in WT$^{fl/fl}$ and YAP/TAZ cKO^{Osx}.

bioRxiv preprint doi: https://doi.org/10.1101/2023.01.20.524918; this version posted January 21, 2023. The copyright holder for this preprint (which was not certified by peer review) is the author/funder, who has granted bioRxiv a license to display the preprint in perpetuity. It is made available under aCC-BY 4.0 International license.

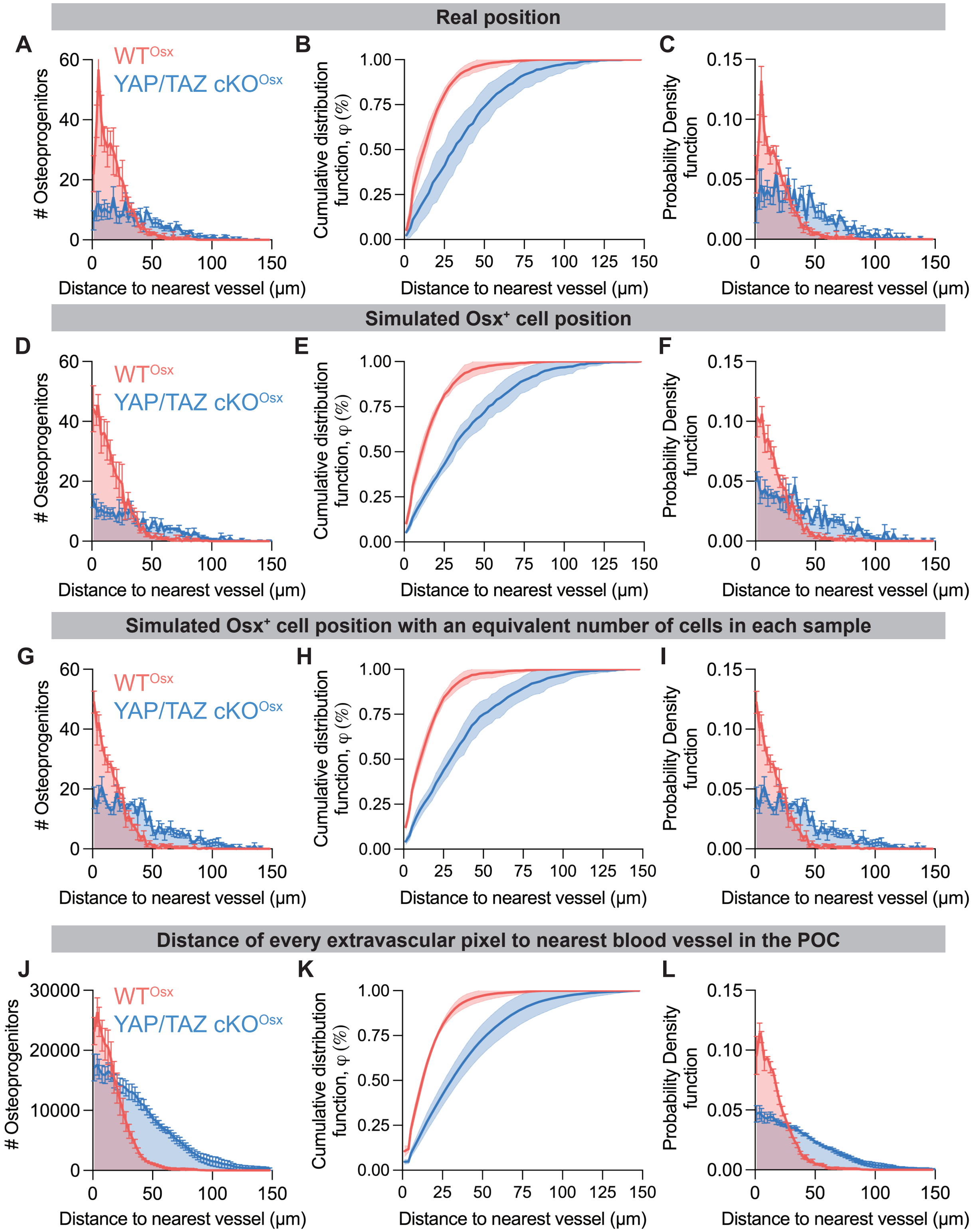

Figure S14. Osx::GFP+ cell proximity to Endomucin⁺ blood vessels and computational varied tests. (**A-C**) (A) histogram, (B) Cumulative distribution function, (C) Probability density function for the real distances from each Osx::GFP+ cell to its nearest blood vessel in the primary ossification center of WT^{Osx} and YAP/TAZ cKO^{Osx}. (**D-F**) (D) histogram, (E) Cumulative distribution function, (F) Probability density function for the first computationally varied condition, in which the position of each Osx::GFP+ cell is randomized within the

bioRxiv preprint doi: https://doi.org/10.1101/2023.01.20.524918; this version posted January 21, 2023. The copyright holder for this preprint (which was not certified by peer review) is the author/funder, who has granted bioRxiv a license to display the preprint in perpetuity. It is made available under aCC-BY 4.0 International license.

extravascular space of its respective primary ossification center. Randomizing the position does not collapse the difference in proximity distribution between WT^{Osx} and YAP/TAZ cKO^{Osx} samples, thus Osx::GFP+ cell position doesn't explain the differences. (**G-I**) (G) histogram, (H) Cumulative distribution function, (I) probability density function for the second computationally varied condition, in which a fixed number (400) of Osx::GFP+ cells are positionally randomized within the extravascular space of its respective primary ossification center. Fixing the number of Osx::GFP+ cells and randomizing their position does not collapse the difference in proximity distribution between WT^{Osx} and YAP/TAZ cKO^{Osx} samples, thus Osx::GFP+ cell density doesn't explain the differences. (**J-L**) (J) histogram, (K) Cumulative distribution function, (L) probability density function for the third computationally varied condition, in which the distance of every extravascular pixel to its nearest vessel was calculated.

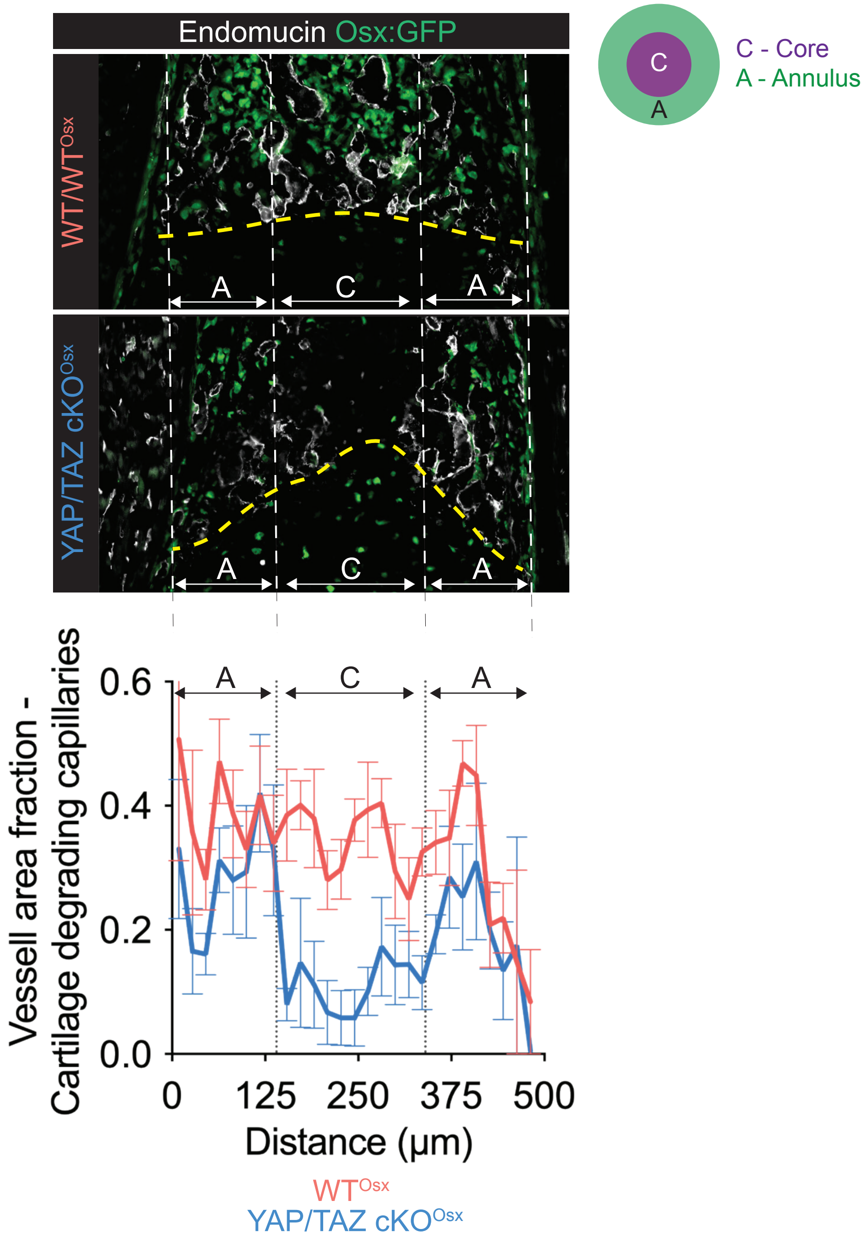

Figure S15. Quantification of the blood vessels within 50µm of the chondro-osseous junction in the core and outer annulus. C – Core. A - Annul

bioRxiv preprint doi: https://doi.org/10.1101/2023.01.20.524918; this version posted January 21, 2023. The copyright holder for this preprint (which was not certified by peer review) is the author/funder, who has granted bioRxiv a license to display the preprint in perpetuity. It is made available under aCC-BY 4.0 International license.

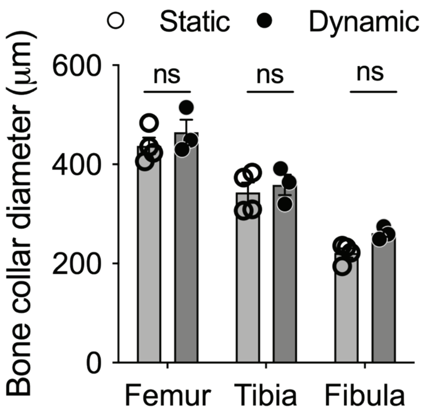

Figure S16. Bone collar diameter of explant C57Bl6 limbs in the cultured in the mechanostimulation bioreactor.

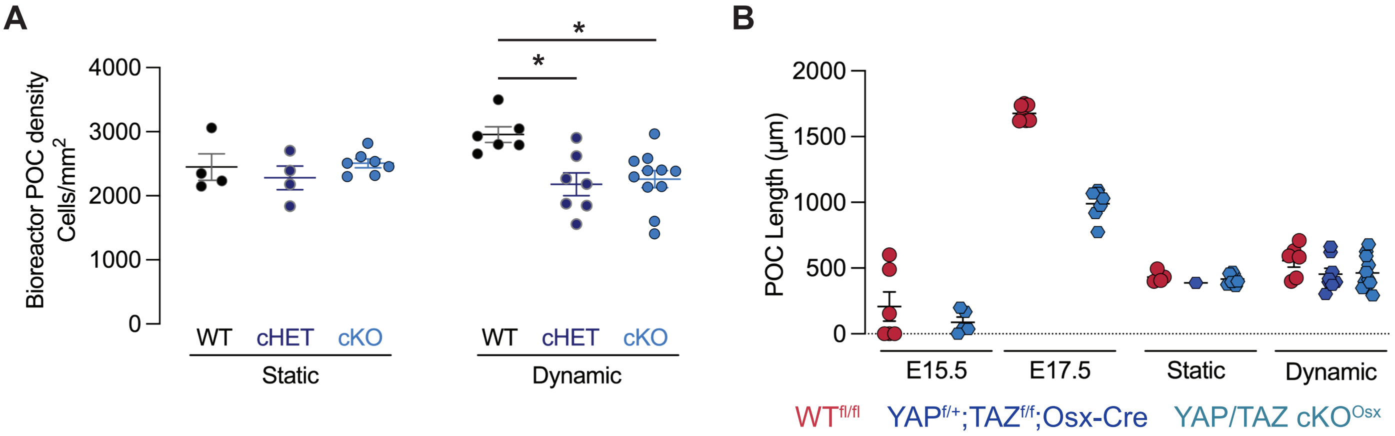

Figure S17. Supplemental data for the genetic Osx-conditional YAP/TAZ deletion bioreactor experiments. (**A**) Primary ossification center (POC) cell density after 6 days of culture in the mechanostimulation bioreactor. (**B**) POC length in development at E15.5 and E17.5 and in E15.5+6d culture under static or dynamic conditions.
